# Sub-plasmalemmal [Ca^2+^]_i_ upstroke in myocytes of the guinea-pig small intestine evoked by muscarinic stimulation: IP_3_R-mediated Ca^2+^ release induced by voltage-gated Ca^2+^ entry

**DOI:** 10.1016/j.ceca.2007.04.012

**Published:** 2008-02

**Authors:** D.V. Gordienko, M.I. Harhun, M.V. Kustov, V. Pucovský, T.B. Bolton

**Affiliations:** a Division of Basic Medical Sciences, Ion Channels and Cell Signalling Centre, St. George's University of London, Cranmer Terrace, London SW17 0RE, UK; b Laboratory of Molecular Pharmacology and Biophysics of Cell Signalling, Bogomoletz Institute of Physiology, 4 Bogomoletz Str., Kiev-024, Ukraine

**Keywords:** Smooth muscle cells, Muscarinic receptors, IP_3_ receptors, Ryanodine receptors, Confocal microscopy, Ca^2+^-induced Ca^2+^ release, E–C coupling

## Abstract

Membrane depolarization triggers Ca^2+^ release from the sarcoplasmic reticulum (SR) in skeletal muscles via direct interaction between the voltage-gated L-type Ca^2+^ channels (the dihydropyridine receptors; VGCCs) and ryanodine receptors (RyRs), while in cardiac muscles Ca^2+^ entry through VGCCs triggers RyR-mediated Ca^2+^ release via a Ca^2+^-induced Ca^2+^ release (CICR) mechanism. Here we demonstrate that in phasic smooth muscle of the guinea-pig small intestine, excitation evoked by muscarinic receptor activation triggers an abrupt Ca^2+^ release from sub-plasmalemmal (sub-PM) SR elements enriched with inositol 1,4,5-trisphosphate receptors (IP_3_Rs) and poor in RyRs. This was followed by a lesser rise, or oscillations in [Ca^2+^]_i_. The initial abrupt sub-PM [Ca^2+^]_i_ upstroke was all but abolished by block of VGCCs (by 5 μM nicardipine), depletion of intracellular Ca^2+^ stores (with 10 μM cyclopiazonic acid) or inhibition of IP_3_Rs (by 2 μM xestospongin C or 30 μM 2-APB), but was not affected by block of RyRs (by 50–100 μM tetracaine or 100 μM ryanodine). Inhibition of either IP_3_Rs or RyRs attenuated phasic muscarinic contraction by 73%. Thus, in contrast to cardiac muscles, excitation–contraction coupling in this phasic visceral smooth muscle occurs by Ca^2+^ entry through VGCCs which evokes an initial IP_3_R-mediated Ca^2+^ release activated via a CICR mechanism.

## 1 Introduction

Smooth muscle cells (SMCs) are stimulated to contract either by depolarization of the cell membrane (electromechanical coupling), often in the form of an action potential (AP), or by activation of a variety of receptors (pharmacomechanical coupling) usually coupled to G-proteins, or by a combination of these mechanisms [1]. Most smooth muscles exhibit voltage-gated Ca^2+^ channels (VGCCs) which are often manifest in the generation of APs, although in many vascular smooth muscles Ca^2+^ is believed to ‘leak’ into the cell through these VGCCs giving rise to ‘sparklets’ [2,3]. The entry of Ca^2+^ into the SMC through VGCCs and by reverse Na^+^/Ca^2+^ exchange [4] is believed to load the Ca^2+^ stores largely in the sarcoplasmic reticulum (SR). From here Ca^2+^ can be released either by a process of Ca^2+^-induced Ca^2+^ release (CICR) through ryanodine receptors (RyRs) upon Ca^2+^ entry during an AP [5] or by the action of inositol 1,4,5 trisphosphate (IP_3_) generated by a G_q/11_-link from an activated stimulant receptor to phospholipase C. The entry of Ca^2+^ through VGCCs is believed to load SR calcium stores which, upon reaching a critical level of loading [6] discharge packets of Ca^2+^ which in the majority of smooth muscles generate transient high local concentrations of sub-plasmalemmal (sub-PM) Ca^2+^ that open Ca^2+^-dependent potassium (BK) channels, present in abundance in the cell membrane of smooth muscles. Bursts of openings of these BK channels are seen as spontaneous transient outward currents (STOCs) in voltage-clamped SMCs [7–9]. In vascular smooth muscles loaded with a fluorescent Ca^2+^ indicator, the transient localized sub-PM increase in Ca^2+^ concentration ([Ca^2+^]_i_) is seen as a flash of emitted light (“spark” [10,11]). The transient BK channel currents evoked have been observed to hyperpolarize the membrane so reducing Ca^2+^ entry through VGCCs, reducing the rate of Ca^2+^ loading of the SR stores, and so reducing tension [11,12]. It seems that Ca^2+^ sparks are a negative feedback mechanism, triggered by store overload, which regulate vascular myocyte tension as they barely increase global [Ca^2+^]_i_, while the high local sub-PM [Ca^2+^]_i_ they create trigger a membrane-potential dependent relaxation of tension through the opening of BK channels [13].

The respective role of SR RyRs and IP_3_ receptors (IP_3_Rs) in the generation of smooth muscle myocyte tension is an area of active investigation. As smooth muscles do not have a sarcomeric organization of their contractile proteins and calcium stores, the sites of Ca^2+^ release and re-storage are necessarily very different from those of striated muscles. In vas deferens and bladder SMCs an AP generates Ca^2+^ release from sub-PM ‘hot spots’ which then generate a cell-wide Ca^2+^ wave presumably by CICR. This is followed by SMC contraction [5]. Activation of stimulant receptors, such as muscarinic receptors in intestinal SMCs, triggers additional spark activity at spontaneously discharging ‘frequent discharge sites’ and recruits additional spark discharge sites [14]. The role of IP_3_Rs seems to be to facilitate CICR within and between RyRs domains [15–18]. In colonic SMCs IP_3_R-evoked Ca^2+^ release did not activate RyRs but RyR blockers inhibited IP_3_R-mediated Ca^2+^ signals [19]. In vascular SMCs of small arteries Lamont and Wier [20] concluded that if RyRs were blocked, cell-wide Ca^2+^ waves could still be evoked by strong activation of adrenoceptors leading to myocyte and vessel contraction; thus CICR release apparently can occur among IP_3_Rs alone although normally, at low levels of adrenoceptor activation RyRs were necessary to trigger IP_3_R dependent Ca^2+^ waves [12]. Thus, at physiologically important levels of tension the interplay of RyR and IP_3_R Ca^2+^ release is important as in visceral smooth muscles. The present work examines the relative roles of RyRs, IP_3_Rs and VGCCs in intracellular Ca^2+^ mobilization in longitudinal intestinal smooth muscle myocytes in response to strong activation of muscarinic receptors. A preliminary account of some of this work has previously been published in an abstract form [21].

## 2 Materials and methods

Experiments were performed on preparations of the longitudinal muscle layer of the guinea-pig ileum: (1) freshly isolated SMCs or (2) freshly dissected strips of smooth muscles (see below). Adult male guinea-pigs (300–500 g) were killed by decapitation after cervical dislocation as approved under Schedule 1 of the UK Animals (Scientific Procedures) Act 1986.

### 2.1 Cell preparation

The longitudinal muscle layer of the ileum was dissected and cut into small pieces, which were placed in Ca^2+^-free physiological saline solution (PSS, see below). The pieces of the tissue were transferred into the same solution supplemented with (mg/ml): protease (Type X) 0.5, collagenase (Type 1A) 1.5, soybean trypsin inhibitor 1 and bovine serum albumin 1, and incubated for 20 min at 37 °C. The pieces of the tissue were then rinsed for 10 min in an enzyme-free Ca^2+^-free solution and triturated with a wide bore glass pipette. Small aliquots of the cell suspension were transferred to the experimental chambers and diluted with PSS composed of (mM): NaCl 120, KCl 6, CaCl_2_ 2.5, MgCl_2_ 1.2, glucose 12, HEPES 10; pH adjusted to 7.4 with NaOH. Experiments on isolated cells were conducted at 22–24 °C within 8 h of cell isolation.

### 2.2 Isometric tension recording

Smooth muscle strips (∼10 mm in length) were dissected from longitudinal layer of guinea-pig ileum, transferred to a homemade organ bath (3 ml volume) and attached to an isometric force transducer at a resting tension load of 5 mN and bathed in the PSS at 37 °C. The output of the force transducer was connected to a custom-made amplifier. The signals were digitized using a Digidata 1320 AD/DA converter (Molecular Devices Co., CA, USA) hosted by a PC running Axoscope 8.0 software (Molecular Devices Co.).

### 2.3 Visualization of [Ca^2+^]_i_ changes

Changes in intracellular concentration of ionized calcium ([Ca^2+^]_i_) in isolated SMCs were imaged using the high-affinity (*k*_d(Ca)_ = 345 nM) fluorescent Ca^2+^ indicator fluo-4, which was loaded by 20-min incubation of the SMCs with 5 μM fluo-4 acetoxymethyl ester (fluo-4 AM) followed by a 40-min wash in PSS to allow time for de-esterification. To minimize SMC contraction, 40 μM of wortmannin was added to the bathing solution 10 min before imaging commenced.

The myocytes were stimulated with 10 μM carbachol (CCh) which was either superfused through the experimental bath or applied as a brief (≤600 ms) pulse through a glass micropipette (located within 100–200 μm of the cell) attached to the outlet of pressure ejector Picospritzer III (Intracel Ltd., UK). Similar application of the control solution (without agonist) had no effect on [Ca^2+^]_i_. In the experiments where the same SMC was stimulated with CCh and caffeine, CCh was superfused through the experimental bath, while 5 mM caffeine was applied through a glass micropipette. Small volumes of antagonists were added to the bath solution in amounts calculated to achieve the indicated concentrations.

In the figures the intensity of fluo-4 fluorescence was normalized to the average fluorescence intensity in the images captured before CCh application and colour coded as indicated by the bars (*F*/*F*_0_). The temporal profiles of CCh-induced [Ca^2+^]_i_ mobilization are illustrated by the plots showing (1) the time course of the normalized fluo-4 fluorescence intensity (*F*/*F*_0_) averaged within multiple sub-PM regions (outlined in the corresponding images) where *F*/*F*_0_ changes were initiated and rose above 2.5 or (2) the time course of *F*/*F*_0_ averaged within the entire confocal optical slice.

### 2.4 Visualization of intracellular calcium stores

Distribution of intracellular calcium stores within isolated SMCs was visualized using the low-affinity (*k*_d(Ca)_ = 42 μM) fluorescent Ca^2+^ indicator fluo-3FF, which was loaded by 90-min incubation of the SMCs with 5 μM fluo-3FF AM followed by 60-min wash in PSS.

### 2.5 Immunostaining of RyRs and IP_3_Rs

Isolated SMCs were fixed by 5–10-min incubation with 4% (w/v) paraformaldehyde. Nonspecific binding was blocked by incubating the SMCs with 3% (w/v) bovine serum albumin (BSA) and 0.3% (w/v) Triton X-100 (a cell permeabilizing agent) for 1 h at room temperature. Primary and secondary antibodies were diluted in PSS supplemented with 3% (w/v) BSA, 0.3% (w/v) Triton X-100, 20 U/ml penicillin and 20 μg/ml streptomycin. To visualize the distribution of IP_3_Rs, we used an IP_3_R type 1-specific antibody, since type 1 IP_3_R was shown to be ubiquitous in various tissues [22]. This antibody was developed (Sigma–Aldrich Co., RBI, Natick, MA, USA) in rabbit and has been shown to selectively recognize type 1 IP_3_Rs in other types of SMCs [23]. RyRs were detected with a monoclonal anti-RyR antibody derived (Sigma–Aldrich) from the 34C hybridoma (produced by the fusion of P3X 63 Ag8.653 myeloma cells and spleen cells from Balb/c mice). This antibody reacts strongly with RyR types 1–3. The SMCs were incubated in the presence of the primary anti-IP_3_R type 1 and anti-RyR antibodies (at 1:300 and 1:480 dilution, respectively) for 16 h at 4 °C. Following a 10-min rinse (four times) in PSS supplemented with 3% (w/v) BSA, primary antibody-specific binding was visualized by incubating SMCs for 2 h at room temperature with Alexa Fluor 488 conjugated to chicken anti-rabbit IgG (1:300 dilution, Invitrogen Ltd., UK) and Alexa Fluor 633 conjugated to goat anti-mouse IgG (1:300 dilution, Invitrogen Ltd., UK). In controls, primary antibodies were omitted from the experimental media during the first (16 h) incubation.

### 2.6 Confocal microscopy

Experimental chambers were placed on the stage of an Axiovert 100 M inverted microscope attached to a LSM 510 laser-scanning unit (Zeiss, Oberkochen, Germany). The *x*–*y* confocal images of fluo-4 fluorescence were acquired at 19–42 Hz using a Zeiss plan-Apochromat 40 × 1.3 N.A. oil-immersion objective. Fluo-4 and fluo-3FF fluorescence was excited by the 488 nm line of a 200 mW argon ion laser (Laser-Fertigung, Hamburg, Germany) and was captured at wavelengths above 505 nm. The pinhole was set to provide a confocal optical slice below 1.2 μm.

To avoid any bleed-through in immunofluorescence experiments, SMCs were double labelled using fluorophores with extremely well separated emission spectra: Alexa Fluor 488 (*E*_m_ = 519 nm) and Alexa Fluor 633 (*E*_m_ = 647 nm). Dual excitation, using the multitrack mode of an LSM 510, was performed by the 488 nm line of a 200 mW argon ion laser and the 633 nm line of a 15 mW helium/neon ion laser, respectively. The emitted fluorescence signal was filtered using 505–550 nm bandpass filter (for the green IP_3_R label) and 650 nm longpass filter (for the red RyR label). The adequacy of the imaging protocol applied to the double-labelled SMCs was confirmed by control experiments on the single-labelled cells.

### 2.7 Electrical recordings

The cell membrane potential was monitored using perforated-patch (200 μg/ml amphotericin B) tight seal recording in the current-clamp mode of an Axopatch 200A (Molecular Devices Co.) patch-clamp amplifier. This allowed low resistance access to the cell while minimally interfering with [Ca^2+^]_i_. The cell was bathed in PSS and dialysed with solution composed of (mM): KCl 120, KH_2_PO_4_ 5, MgSO_4_ 5, Na_2_ATP 5, Li_2_GTP, Hepes 10; pH was adjusted to 7.4 with KOH. Recording of the cell membrane potential was synchronized with confocal imaging using a TTL synchronizing pulse generated by the confocal scanner at the beginning of the time series protocol.

Membrane currents were recorded using the conventional whole-cell patch clamp technique using an Axopatch 200A or a Multiclamp 700A patch-clamp amplifier (Molecular Devices Co.). Liquid junction potential was nulled with the offset circuit of the amplifier before seal formation. Pipette and cell capacitance and series resistance were compensated electronically using corresponding amplifier controls. No electronic compensation for the leakage conductance was introduced. The electrical signals were filtered at 1 kHz (−3 dB frequency) by a four-pole low-pass Bessel filter. Voltage protocols were generated and electrical signals were digitized at 5 kHz using a DigiData 1200 or a Digidata 1322A hosted by a Pentium PC running either pCLAMP 6.0 or pClamp 8.2 software (Molecular Devices Co.).

Muscarinic cationic current (mI_cat_) activated by external application of 10 μM CCh (see above) was recorded at holding potential of −50 mV. Background holding current was measured before CCh application. In these experiments, the composition of extracellular solution was (mM): CsCl 120, CaCl_2_ 2.5, MgCl_2_ 1.2, glucose 12, Hepes 10 (pH 7.4 adjusted with CsOH) and the composition of the pipette solution was (in mM): CsCl 80, MgATP 1, creatine 5, glucose 5, Hepes 10 (pH adjusted to7.4 with CsOH). To unmask any direct effect of the IP_3_R inhibitor 2-APB on mI_cat_, the effect of 2-APB on mI_cat_ mediated via inhibition of IP_3_R-mediated Ca^2+^ release was eliminated by clamping [Ca^2+^]_i_ at 100 nM with 4.6 mM CaCl_2_/ 10 mM BAPTA buffer added to the pipette solution [24].

Voltage-gated Ca^2+^ currents were evoked by 500-ms voltage steps to 0 mV applied from a holding potential of −80 mV every 30 s. In these experiments, the composition of the extracellular solution was (mM): NaCl 135, CsCl 6, CaCl_2_ 2.5, MgCl_2_ 1.2, glucose 12, Hepes 10 (pH 7.4 adjusted with NaOH) and the composition of the pipette solution was (in mM): CsCl 126, MgSO_4_ 5, Na_2_ATP 5, Li_2_GTP 1 (pH adjusted to7.4 with CsOH and [Ca^2+^]_i_ was clamped at 20 nM with 2.6 mM CaCl_2_/10 mM EGTA buffer).

### 2.8 Reagents

Protease (Type X), collagenase (type 1A), soybean trypsin inhibitor (Type II-S), bovine serum albumin, adenosine 5′ triphosphate (ATP, magnesium salt), guanosine 5’ triphosphate (GTP, disodium salt), creatine, *N*-2-hydroxyethylpiperazine-*N*′-2-ethanesulphonic acid (HEPES), 1,2-bis(2-aminophenoxy)-ethane-*N*,*N*,*N*′,*N*′-tetraacetic acid (BAPTA), ethylene glycol-bis(2-aminoethylether)-*N*,*N*,*N*′,*N*′-tetraacetic acid (EGTA), carbamylcholine chloride (carbachol), 1,3,7-trimethylxanthine (caffeine), dimethyl sulfoxide (DMSO), paraformaldehyde and Triton X-100 were obtained from Sigma Chemical Co., Poole, Dorset, UK. Fluo-4 acetoxymethyl ester, Alexa Fluor 488 conjugated chicken anti-rabbit IgG (H + L), Alexa Fluor 633 conjugated goat anti-mouse IgG (H + L) and pluronic F-127 were obtained from Invitrogen Ltd., Paisley, UK. Fluo-3FF acetoxymethyl ester was from TefLabs, Austin, Texas, USA. All other chemicals were from BDH Laboratory Supplies (AnalaR grade), Pool, UK.

### 2.9 Data analysis

Image processing was carried out using an Indy workstation (Silicon Graphic Inc., Mountain View, CA, USA) with custom routines written in IDL (Research Systems Inc., Boulder, CO, USA). The final figures were produced using MicroCal Origin (MicroCal Software Inc., Northampton, MA, USA) and CorelDraw 7.0 (Corel Corporation, Canada). Where appropriate, data are expressed as mean values ± S.E.M. for the number of cells (*n*) analysed. Comparative analysis of the data groups was performed using Student's *t*-test for paired or unpaired samples, as appropriate, with the threshold for statistical significance set at the 0.05 level.

## 3 Results

### 3.1 Action potential discharge following muscarinic receptor activation is coupled to a sub-PM [Ca^2+^]_i_ upstroke (SPCU)

**Fig. 1:**
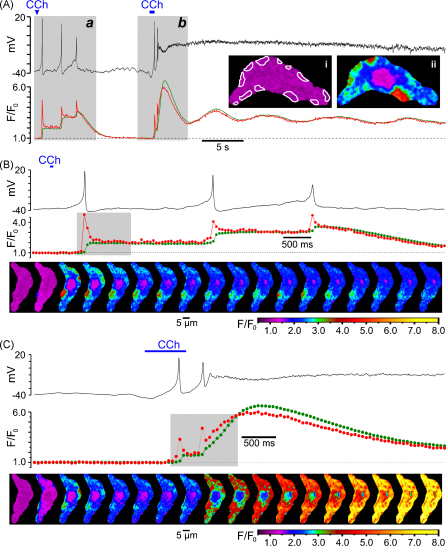
Carbachol (CCh)-induced action potentials are associated with sub-plasmalemmal (sub-PM) [Ca^2+^]_i_ upstroke (SPCU). (A) The record of the cell membrane potential (black trace) is superimposed on the time course of the normalized intensity of fluo-4 fluorescence averaged (red trace) within 12 sub-PM regions outlined in (i) and (green trace) within the total confocal optical slice of SMC. The outlined regions in this and in all subsequent figures were the sites of CCh-induced Ca^2+^ wave initiation, as illustrated in (ii). The response of the SMC was triggered by 10 μM CCh applied twice from a glass micropipette: as 60-ms pulse (Aa and B) and as 600-ms pulse (Ab and C). The confocal images were acquired 54 ms apart. The fluorescence intensity was normalized to the average fluorescence intensity in a series of images captured before CCh application and colour coded as indicated (*F*/*F*_0_). Two periods of interest highlighted (grey background) in (A) are presented on an expanded time scale in (B and C). In (B and C) the gallery below the plot shows 18 sequential confocal images (after rotation by 90°) captured during the period highlighted in the corresponding plot of the normalized fluorescence signal. See also video clips in supplementary material on line.

The dynamics of the change in intracellular Ca^2+^ concentration ([Ca^2+^]_i_) following muscarinic receptor activation were related to the changes in the cell membrane potential (*V*_m_). SMCs freshly isolated from the guinea-pig ileum were preloaded with the high affinity Ca^2+^ indicator fluo-4 and bathed in PSS (see Section 2). High speed *x*–*y* confocal Ca^2+^ imaging (acquisition rate varied between 19 and 42 Hz) was combined with recording of *V*_m_ in perforated-patch configuration to minimally perturb endogenous [Ca^2+^]_i_ (*n* = 4). Muscarinic receptors were stimulated by brief applications of 10 μM carbachol (CCh) through a glass micropipette positioned within 100–200 μm of the cell surface. This revealed that the discharge of each action potential (AP) following muscarinic receptor activation was associated with a [Ca^2+^]_i_ transient which was initiated by an abrupt increase of [Ca^2+^]_i_ at multiple sub-plasmalemmal (sub-PM) regions. The dynamics of [Ca^2+^]_i_ changes in these sub-PM regions of initiation was therefore analysed. In the example shown in Fig. 1, the first 60-ms application of 10 μM CCh triggered three APs but no sustained membrane depolarization (probably because equilibrium CCh concentration near the cell membrane was not achieved). Even though after each action potential the cell membrane was repolarized to a resting level (black trace, Fig. 1A and B), each AP was associated with a stepwise increase of the global [Ca^2+^]_i_ (green trace Fig. 1A and B). The second 600-ms application of 10 μM CCh caused membrane depolarization, which was associated with increased frequency of APs. Within 1.5 s depolarization became so extreme that AP discharge ceased (black trace, Fig. 1A and C). The global [Ca^2+^]_i_ response caused by the 600-ms CCh application (green trace Fig. 1A and C) consisted of an initial large-amplitude transient followed by a sustained phase showing small amplitude oscillations. In both cases, however, each AP was coupled to a very brief abrupt [Ca^2+^]_i_ transient (red trace, Fig. 1) at multiple sub-PM regions (illustrated by (ii) and outlined in (i), Fig. 1A). The spatio-temporal profiles of intracellular Ca^2+^ mobilizations associated with the first AP following the first CCh application, and with the two APs following the second CCh application, are illustrated by two galleries of 18 sequential confocal images (Fig. 1B and C, respectively). Comparing the images in the galleries revealed a remarkable constancy of the positions of the sites of initiation of Ca^2+^ mobilization associated with each AP. The lack of spatial uniformity suggests that rather than Ca^2+^ entry through voltage-gated Ca^2+^ channels (VGCCs) these signals reflect Ca^2+^ release from non-uniformly distributed sub-PM Ca^2+^ stores. Because of their location and robust onset kinetics, we refer to these events hereafter as the “sub-PM [Ca^2+^]_i_ upstroke (SPCU)”.

**Fig. 2:**
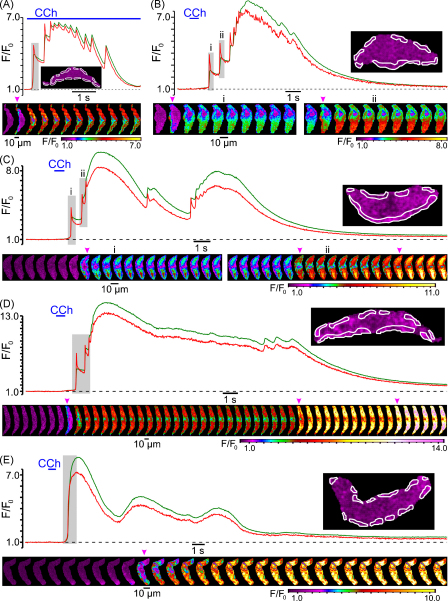
Spatio-temporal patterns of CCh-induced [Ca^2+^]_i_ transients. The *x*–*y* confocal Ca^2+^ imaging was performed at 32 Hz (A and B), 40 Hz (C), 44 Hz (D) and 30 Hz (E). For each cell, the time course plot of the normalized fluo-4 fluorescence intensity was averaged (red trace) within sub-PM regions of interest where Ca^2+^ waves (induced by 10 μM CCh) were initiated (insets), and (green trace) within the total confocal optical slice of the SMC. The galleries below the plots show sequential confocal images (after rotation by 90°) captured during the periods highlighted by grey background in the plots. Magenta arrowheads in the galleries indicate sub-PM [Ca^2+^]_i_ upstrokes.

Spatio-temporal patterns of CCh-induced [Ca^2+^]_i_ mobilization in non-patched SMCs are illustrated by Fig. 2 showing the results obtained in 5 different cells. Muscarinic receptors were activated by 10 μM CCh either superfused through the experimental bath (Fig. 2A) or applied to the cell as a 600-ms pulse through a glass micropipette (Fig. 2B–E). In all plots, the green traces show the temporal profile of the global [Ca^2+^]_i_ changes, while red traces show the dynamics of [Ca^2+^]_i_ changes averaged at multiple sub-PM regions (outlined in the images; insets on the plots) where CCh-induced [Ca^2+^]_i_ transients were initiated. The galleries show sequential confocal images of fluo-4 fluorescence acquired during the periods marked by a grey background in the plots, respectively. In all cases CCh-induced [Ca^2+^]_i_ transients were initiated by a SPCU (depicted by the arrowheads on the galleries). Muscarinic receptor activation evoked a SPCU independently of the extent of myocyte contraction (*n* = 147), suggesting that this phenomenon is not a result of the increase in the local density of Ca^2+^-release units, which could be caused by change in SMC geometry. In the vast majority of cases (97%) two phases can be clearly distinguished in the Ca^2+^ responses to CCh: (1) an initial high-amplitude [Ca^2+^]_i_ transient and (2) a delayed increase of global [Ca^2+^]_i_ characterized by a smaller amplitude and a tendency to oscillation.

### 3.2 Genesis of SPCU depends on both voltage-gated Ca^2+^ entry and Ca^2+^ release from the SR

**Fig. 3:**
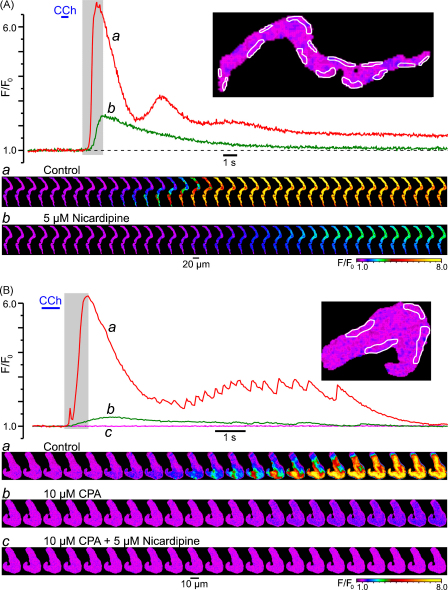
CCh-induced SPCU depends on both voltage-gated Ca^2+^ entry and Ca^2+^ release from intracellular stores: effect of block of voltage-gated Ca^2+^ channels with 5 μM nicardipine (A) and depletion of intracellular Ca^2+^ stores with 10 μM CPA (B). The imaging was performed at 26 Hz (A) and 23 Hz (B). The plot shows the time course of the normalized fluo-4 fluorescence intensity averaged within sub-PM regions (outlined) in control (a), after incubation with 5 μM nicardipine or 10 μM CPA (b) and after incubation with 5 μM nicardipine in the presence of 10 μM CPA (c). A 10-min period was allowed between subsequent 600-ms pulses of 10 μM CCh. The galleries below the plots show sequential confocal images (after rotation by 90°) captured during the highlighted periods.

Muscarinic cationic channels in gastro-intestinal smooth muscles have very low, if any, permeability for Ca^2+^ [25–27], and the major physiological role of muscarinic cationic current (mI_cat_) is to depolarize the cell membrane and to trigger the opening of voltage-gated Ca^2+^ channels (VGCCs), pharmacological blockade of which virtually completely abolishes muscarinic contractile responses [28,29]. We therefore tested the effect of VGCC block on CCh-induced [Ca^2+^]_i_ mobilization. In this and all subsequent experiments we analysed the dynamics of [Ca^2+^]_i_ changes at multiple sub-PM regions of initiation of CCh-induced [Ca^2+^]_i_ mobilization. In control, the response to CCh was initiated by a SPCU (Fig. 3A, red trace) and then rapidly propagated through the entire cell volume (Fig. 3A, gallery a). The initial [Ca^2+^]_i_ transient was followed by a lower amplitude sustained phase with two [Ca^2+^]_i_ oscillations (Fig. 3A, red trace). Block of voltage-gated Ca^2+^ channels with 5 μM nicardipine (30-s incubation) eliminated the SPCU (Fig. 3A, gallery b) and substantially attenuated, but did not abolish, both the initial and delayed phase of the CCh-induced [Ca^2+^]_i_ transient (Fig. 3A, green trace). In the presence of nicardipine, the peak of CCh-induced [Ca^2+^]_i_ transient (green trace) was reduced by 60%, time-to-peak was increased from 0.8 to 1.3 s and the Ca^2+^ wave propagated more slowly than in control (Fig. 3A, gallery b). These results suggest that: (1) the SPCU requires voltage-gated Ca^2+^ entry and (2) Ca^2+^ entry through VGCCs is not the only source of Ca^2+^ upon muscarinic [Ca^2+^]_i_ mobilization.

To evaluate the contribution of Ca^2+^ release from intracellular stores to CCh-induced [Ca^2+^]_i_ mobilization, the effect of Ca^2+^ store depletion was tested. In control, CCh induced a rapidly propagating [Ca^2+^]_i_ wave (Fig. 3B, gallery a) which was initiated by a SPCU (regions of initiation are outlined in the image, Fig. 3B). The initial [Ca^2+^]_i_ transient was followed by a lower amplitude sustained phase showing multiple small-amplitude but high-frequency oscillations (Fig. 3B, red trace). A 10-min incubation of the ileal SMCs with 10 μM cyclopiazonic acid (CPA), a reversible inhibitor of the sarco/endoplasmic reticulum Ca^2+^-ATPase (SERCA), resulted in complete depletion of the intracellular Ca^2+^ stores in this cell type, as we have previously demonstrated using flash release of “caged” IP_3_ preloaded into the cells through the patch pipette [24]. Depletion of Ca^2+^ stores with CPA treatment eliminated the SPCU, reduced the peak amplitude of the initial CCh-induced [Ca^2+^]_i_ transient by 80%, increased time-to-peak from 0.7 to 1.4 s and virtually completely abolished the delayed phase of the response (Fig. 3B, green trace). With Ca^2+^ stores depleted, the residual [Ca^2+^]_i_ response was spatially uniform (Fig. 3B, gallery b), consistent with Ca^2+^ entry through VGCCs. This was further confirmed when subsequent block of VGCCs with 5 μM nicardipine (while keeping Ca^2+^ stores depleted) completely abolished [Ca^2+^]_i_ mobilization in response to CCh (Fig. 3B, magenta trace and gallery c). This also directly demonstrates that muscarinic cationic channels in SMCs of the guinea-pig ileum are virtually impermeable to Ca^2+^.

Summing up, the above results show that both Ca^2+^ entry through VGCCs and Ca^2+^ release from intracellular Ca^2+^ stores contribute to the SPCU and full scale [Ca^2+^]_i_ mobilization in response to muscarinic stimulation.

### 3.3 SPCU results from IP_3_R-mediated Ca^2+^ release facilitated by Ca^2+^ entry through VGCCs

**Fig. 4:**
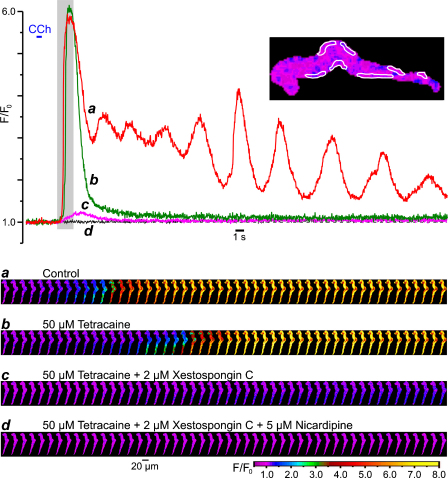
Components of the CCh-induced [Ca^2+^]_i_ transient: successive cumulative inhibitions of RyRs, IP_3_Rs and VGCCs. The fluo-4 loaded SMC was stimulated with 10 μM CCh (600-ms pulse) and imaged at 31 Hz. A 10-min period was allowed between CCh pulses. The time course of the normalized fluo-4 fluorescence averaged within seven sub-PM regions (outlined) was plotted in control (a), and following successive cumulative inhibitions by 50 μM tetracaine (b), 2 μM xestospongin C (c) and 5 μM nicardipine (d). The galleries below the plot show sequential images (after rotation by 90°) taken during the highlighted period.

To examine what type of the SR Ca^2+^ release channels is involved in SPCU and what the mechanisms underlie the subsequent transient and delayed phases of CCh-induced [Ca^2+^]_i_ mobilization, we studied the effects of successive cumulative inhibitions of RyRs, IP_3_Rs and VGCCs on Ca^2+^ responses to CCh. The SMCs were incubated for 10 min with 50–100 μM tetracaine [30–32] to block RyRs and with 2 μM xestospongin C [16,33,34] to block IP_3_Rs. In the example shown in Fig. 4, control response to CCh consisted of a [Ca^2+^]_i_ transient initiated by a SPCU and followed by numerous [Ca^2+^]_i_ oscillations of gradually decreasing amplitude (Fig. 4, red trace and gallery a). Nevertheless, block of RyRs by tetracaine did not abolish the SPCU and even slightly (5%) augmented the initial [Ca^2+^]_i_ transient, but abolished the delayed phase of the response to CCh and the [Ca^2+^]_i_ oscillations (Fig. 4, green trace and gallery b). The slight increase of the initial [Ca^2+^]_i_ transient may result from the increase in the SR Ca^2+^ load following tetracaine treatment [31]. The overall effect of tetracaine on CCh-induced [Ca^2+^]_i_ mobilization implies that RyRs play little role, if any, in the SPCU, but are important for the sustained response to CCh and in [Ca^2+^]_i_ oscillations.

A subsequent incubation of the myocyte with 2 μM xestospongin C (in the presence of tetracaine) abolished the SPCU, inhibited the initial [Ca^2+^]_i_ transient by 95% and increased the time-to-peak from 1 to 2.5 s (Fig. 4, magenta trace c). The remaining CCh-induced [Ca^2+^]_i_ transient was characterized by a slow and spatially uniform rising phase, consistent with Ca^2+^ entry through VGCCs (Fig. 4, gallery c). This observation indicates that the SCPU strongly depends on the ability of IP_3_Rs to release Ca^2+^.

Subsequent block of VGCCs with 5 μM nicardipine (1-min incubation) completely abolished the residual response to CCh (Fig. 4, black trace and gallery d), thus confirming the idea that the uniform transient increase of [Ca^2+^]_i_ triggered by CCh in the presence of tetracaine and xestospongin C was caused by Ca^2+^ entry through VGCCs.

**Fig. 5:**
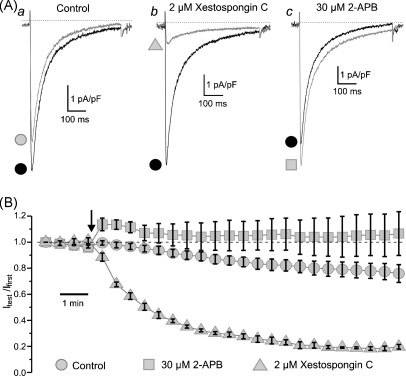
Effect of xestospongin C and 2-APB on voltage-gated Ca^2+^ current (*I*_Ca_). *I*_Ca_ was recorded under whole-cell voltage clamp in response to 500-ms steps to 0 mV, applied every 30 s from *V*_h_ = −80 mV, in Cs^+^/Na^+^-containing solutions (see Section 2). The traces (A) show *I*_Ca_ evoked by the first step (black) and the step applied 10 min after (grey) in control (a), in 2 μM xestospongin C (b) and in 30 μM 2-APB (c). The normalized peak *I*_Ca_ (*I*_test_/*I*_first_) is plotted over time (B) in control (circle, *n* = 7), in 2 μM xestospongin C (triangle, *n* = 4) and in 30 μM 2-APB (square, *n* = 5). The drug application moment is depicted by the arrow.

It is known, however, that commercially available inhibitors of IP_3_R-mediated Ca^2+^-release, namely xestospongin C and 2-aminoethoxy-diphenylborate (2-APB), may affect some other mechanisms of intracellular Ca^2+^ homeostasis. Since the key event in muscarinic [Ca^2+^]_i_ mobilization seems to be the Ca^2+^ entry through VGCCs, we tested the effect of 2 μM xestospongin C and 30 μM 2-APB on the voltage-gated Ca^2+^ current (*I*_Ca_) evoked by a 500-ms voltage steps from −80 to +0 mV under whole-cell voltage clamp conditions (Fig. 5). The currents were recorded in Cs^+^/Na^+^-containing solutions (see Section 2) while the pipette solution was supplemented with 5 mM ATP and 1 mM GTP to minimize rundown of *I*_Ca_. The peak amplitude of *I*_Ca_ (Fig. 5A) evoked by repetitive (applied at 30-s intervals) voltage steps was normalized to the peak amplitude of *I*_Ca_ evoked by the first voltage step (*I*_test_/*I*_first_) and plotted over time (Fig. 5B) in control (*n* = 7) and following application of 2 μM xestospongin C (*n* = 4) or 30 μM 2-APB (*n* = 5). This revealed that after allowing for *I*_Ca_ rundown, the reduction of the current amplitude by a further 74% during 10 min (from *I*_test_/*I*_first_ = 0.779 ± 0.058 in control to *I*_test_/*I*_first_ = 0.184 ± 0.013 in xestospongin C; significant difference: *p* = 0.00018) can account for the inhibitory effect of xestospongin C on VGCCs. In contrast, 2-APB during the same period augmented *I*_Ca_ (taking into account the *I*_Ca_ rundown) on average by 35% (from *I*_test_/*I*_first_ = 0.779 ± 0.058 in control to *I*_test_/*I*_first_ = 1.056 ± 0.159 in 2-APB; not significantly different: *p* = 0.073). Thus, the inhibitory effect of 2 μM xestospongin C on CCh-induced [Ca^2+^]_i_ mobilization may partially (since CCh-induced [Ca^2+^]_i_ transient was not abolished by xestospongin C but was eliminated by subsequent block of VGCCs with nicardipine; Fig. 4) result from inhibition of VGCCs by this compound.

**Fig. 6:**
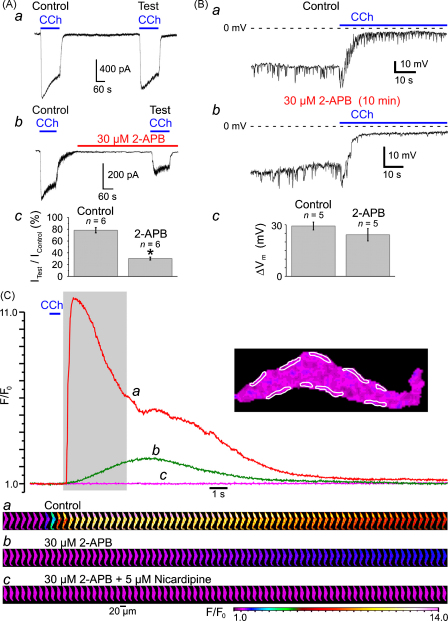
Effect of 2-APB on (A) muscarinic cationic current (mI_cat_), (B) CCh-induced membrane depolarization and (C) CCh-induced [Ca^2+^]_i_ mobilization. (A) mI_cat_ was activated at −50 mV by 10 μM CCh in SMC with [Ca^2+^]_i_ clamped at 100 nM with Ca^2+^/BAPTA buffer and was recorded in Cs^+^-containing solutions (see Section 2). Peak mI_cat_ (a) triggered by the second CCh application (Test) was related to that triggered by the first CCh application (Control). 30 μM 2-APB reduced the peak mI_cat_ (b) on average (*n* = 6) by 61.5% (c). The symbol (*) shows the significant difference (*p* < 0.0002) between control and 2-APB. Application of 10 μM CCh depolarized the cell membrane from −33 ± 2 mV to −4 ± 1 mV in control (Ba) and from −29 ± 4 mV to −5 ± 1 mV (*n* = 5) in 30 μM 2-APB (Bb). Summarized in (Bc). The fluo-4-loaded SMC was stimulated with 600-ms pulses of 10 μM CCh at 10-min intervals and imaged at 39 Hz (C). The time course of the normalized fluo-4 fluorescence averaged within nine sub-PM regions (outlined) was plotted in control (a), after incubation with 30 μM 2-APB (b) and after incubation with 5 μM nicardipine in the presence of 30 μM 2-APB (c). The galleries below the plot show sequential images (after rotation by 90°) taken during the period highlighted in the plot.

Taking into account that activation of VGCCs in response to muscarinic receptor activation is caused by depolarization evoked by mI_cat_, and thus will not occur if the muscarinic cationic channels are blocked, the effects of 30 μM 2-APB on mI_cat_ (Fig. 6A) were tested. The current induced by 10 μM CCh was recorded under whole-cell voltage clamp at a holding potential of −50 mV using Cs^+^-containing pipette and bathing solutions (see Section 2). Since mI_cat_ is Ca^2+^-sensitive [24,26,35–37], to eliminate the effect of 2-APB caused by inhibition of IP_3_R-mediated Ca^2+^ release, [Ca^2+^]_i_ was clamped at 100 nM (see Section 2). Each SMC was stimulated twice with a 10-min interval between subsequent CCh applications. The response to the second CCh application (Test) was related to the response to the first CCh application (Control). In control external solution, the peak amplitude of the test mI_cat_ (Fig. 6Aa) constituted on average 78 ± 4% (*n* = 6) of the control mI_cat_ (Fig. 6Ac). Incubation with 30 μM 2-APB for 8 min prior to the second CCh application reduced the peak amplitude of the test mI_cat_ (Fig. 6Ab) to the 30 ± 2% (*n* = 6) of the control mI_cat_ (Fig. 6Ac). Thus, 30 μM 2-APB caused inhibition of mI_cat_ by 61.5% (significant difference: *p* = 0.00019).

However, in terms of CCh-induced [Ca^2+^]_i_ mobilization, the amplitude of mI_cat_ is not of as much importance (since this current is not conducted by Ca^2+^ under quasi-physiological conditions, see above) as the level of the cell membrane depolarization caused by this current. Indeed, any ionic current cannot change the cell membrane potential (*V*_m_) beyond the level of the reversal potential of the current. In the case when the input resistance of the cell is relatively high (such as at resting condition) even a small amplitude current can produce a substantial shift in *V*_m_. We therefore tested the effect of 30 μM 2-APB on CCh-induced membrane depolarization (Fig. 6B). The SMCs were bathed in PSS and dialysed with K^+^/Na^+^-containing solution (see Section 2). Application of 10 μM CCh shifted *V*_m_ from −33 ± 2 mV to −4 ± 1 mV (*n* = 5) in control (Ba) and from −29 ± 4 mV to −5 ± 1 mV (*n* = 5) in 30 μM 2-APB (Bb), (*n* = 5). The values of the magnitude (Δ*V*_m_) of membrane depolarization (Bc) caused by CCh in control (29 ± 2 mV) and in 2-APB (24 ± 4 mV), as well as extent of the CCh-induced depolarization in control and in 2-APB were not significantly different (*p* = 0.23 and *p* = 0.52, respectively). This validates 2-APB as pharmacological tool for study of the role of IP_3_Rs in CCh-induced [Ca^2+^]_i_ mobilization.

**Fig. 7:**
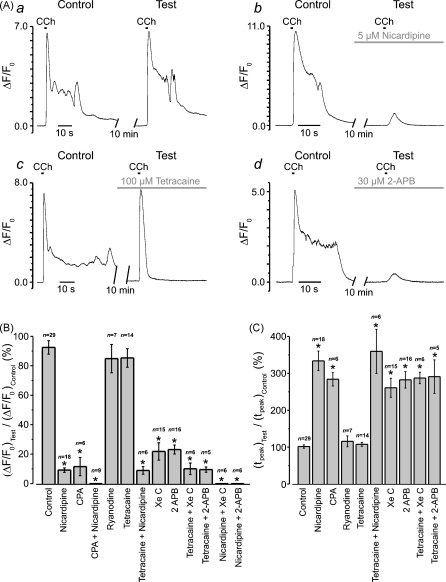
Summary of the effects of VGCC/SERCA/RyR/IP_3_R inhibitors on the initial phase of CCh-induced [Ca^2+^]_i_ transient. Experimental protocol is illustrated in (A). The fluo-4 loaded SMCs were stimulated with 600-ms pulses of 10 μM CCh applied with a10 min interval. The response (Δ*F*/*F*_0_ averaged at multiple sub-PM regions) to the second CCh application (Test) was related to the response to the first CCh application (Control). The Test response was obtained either in the control (a), or following incubation with a drug (b–d) or a combination of several drugs. Two parameters were examined and summarized: (B) relative change in peak amplitude (Δ*F*/*F*_0_)_Test_/(Δ*F*/*F*_0_)_Control_, and (C) relative change in time-to-peak, (*t*_peak_)_Test_/(*t*_peak_)_Control_. The symbol (*) shows the significant difference (*p* < 0.0000002) between the parameters in control external solution and in the presence of drug (or drug combination), as indicated.

Inhibition of IP_3_Rs by 10-min incubation of the SMC with 30 μM of 2-APB abolished the SPCU, inhibited the CCh-induced [Ca^2+^]_i_ transient by 79% and increased time-to-peak from 0.5 to 4.5 s (Fig. 7C, green trace). Subsequent block of VGCCs with 5 μM nicardipine (30-s incubation) completely abolished the residual response to CCh (Fig. 6C, magenta trace and gallery c). This observation confirmed our conclusion that following muscarinic receptor activation Ca^2+^ entry through VGCCs facilitates the initial IP_3_R-mediated Ca^2+^ release at sub-PM regions leading to a SPCU, which in turn engages full scale [Ca^2+^]_i_ mobilization.

The effects of the Ca^2+^ store depletion, inhibition of VGCCs, RyRs and IP_3_Rs on the CCh-induced [Ca^2+^]_i_ transient initiated by SPCU are summarized in Fig. 7. In all experiments the fluo-4 loaded SMCs were stimulated with 600-ms pulses of 10 μM CCh, which were applied to the same cell at least twice with a 10-min interval between subsequent CCh applications (Fig. 7A). The increase in the normalized intensity of fluo-4 fluorescence (Δ*F*/*F*_0_) averaged at multiple sub-PM regions of initiation of the CCh-induced [Ca^2+^]_i_ transient was then plotted over time. The response to the succeeding CCh application (Test) was related to the response to the first CCh application (Control). The test response was obtained either in the control external solution (to evaluate reproducibility of CCh-induced [Ca^2+^]_i_ transients) or following a 10-min incubation of the SMC with a drug or combination of several drugs. When the effect of VGCC block on muscarinic [Ca^2+^]_i_ mobilization was tested, nicardipine was applied 30 s–1 min before the pulse of CCh to minimize possible effect of VGCC block on the SR Ca^2+^ load. Two parameters were examined and are summarized in the bar diagram plots: (1) relative change in peak amplitude (Δ*F*/*F*_0_)_Test_/(Δ*F*/*F*_0_)_Control_ (Fig. 7B) and (2) relative change in time-to-peak, (*t*_peak_)_Test_/(*t*_peak_)_Control_ (Fig. 7C). This revealed that: (i) in control external solution, the peak amplitude of the test response (Fig. 7Aa) constituted on average 92 ± 5% (*n* = 29) of the control response (Fig. 7B), while time-to-peak was on average 102 ± 5% of that in control response (Fig. 7C); (ii) block of RyRs with either 100 μM ryanodine ((Δ*F*/*F*_0_)_Test_/(Δ*F*/*F*_0_)_Control_ = 84 ± 10%, (*t*_peak_)_Test_/(*t*_peak_)_Control_ = 116 ± 15%, *n* = 7) or with 50–100 μM tetracaine ((Δ*F*/*F*_0_)_Test_/(Δ*F*/*F*_0_)_Control_ = 85 ± 6%, (*t*_peak_)_Test_/(*t*_peak_)_Control_ = 108 ± 5%, *n* = 14) had no significant effect on either amplitude (*p* = 0.47 and *p* = 0.37, respectively) or kinetics (*p* = 0.25 and *p* = 0.44, respectively) of the initial [Ca^2+^]_i_ transient; (iii) depletion of intracellular Ca^2+^ stores with 10 μM CPA ((Δ*F*/*F*_0_)_Test_/(Δ*F*/*F*_0_)_Control_ = 11.5 ± 6%, (*t*_peak_)_Test_/(*t*_peak_)_Control_ = 284 ± 18%, *n* = 6) reduced the peak amplitude by 87.5% (*p* = 1.3 × 10^−7^) and increased time-to-peak by 178% (*p* = 1.7 × 10^−15^); (iv) block of VGCCs with 5 μM nicardipine ((Δ*F*/*F*_0_)_Test_/(Δ*F*/*F*_0_)_Control_ = 9 ± 1%, (*t*_peak_)_Test_/(*t*_peak_)_Control_ = 334 ± 27%, *n* = 18) reduced the peak amplitude by 90% (*p* = 5.7 × 10^−18^) and increased time-to-peak by 227% (*p* = 5.2 × 10^−14^); (v) simultaneous block of VGCCs and RyRs with 5 μM nicardipine/50–100 μM tetracaine ((Δ*F*/*F*_0_)_Test_/(Δ*F*/*F*_0_)_Control_ = 9 ± 3%, (*t*_peak_)_Test_/(*t*_peak_)_Control_ = 360 ± 60%, *n* = 6) reduced the peak amplitude by 90% (*p* = 2.2 × 10^−9^) and increased time-to-peak by 253% (*p* = 8.7 × 10^−11^); (vi) block of IP_3_Rs with 2 μM xestospongin C ((Δ*F*/*F*_0_)_Test_/(Δ*F*/*F*_0_)_Control_ = 22 ± 6%, (*t*_peak_)_Test_/(*t*_peak_)_Control_ = 261 ± 26%, *n* = 15) reduced the peak amplitude by 76% (*p* = 1.2 × 10^−11^) and increased time-to-peak by 159% (*p* = 3.3 × 10^−10^); block of IP_3_Rs with 30 μM 2-APB ((Δ*F*/*F*_0_)_Test_/(Δ*F*/*F*_0_)_Control_ = 23 ± 3%, (*t*_peak_)_Test_/(*t*_peak_)_Control_ = 283 ± 22%, *n* = 16) reduced the peak amplitude by 75% (*p* = 2.0 × 10^−13^) and increased time-to-peak by 177% (*p* = 3.6 × 10^−13^); (vii) simultaneous block of RyRs and IP_3_Rs with 50–100 μM tetracaine / 2 μM xestospongin C ((Δ*F*/*F*_0_)_Test_/(Δ*F*/*F*_0_)_Control_ = 10 ± 4%, (*t*_peak_)_Test_/(*t*_peak_)_Control_ = 287 ± 16%, *n* = 6) reduced the peak amplitude by 89% (*p* = 3.4 × 10^−9^) and increased time-to-peak by 181% (*p* = 2.1 × 10^−16^); (viii) simultaneous block of RyRs and IP_3_Rs with 50–100 μM tetracaine / 30 μM 2-APB ((Δ*F*/*F*_0_)_Test_/(Δ*F*/*F*_0_)_Control_ = 9 ± 2%, (*t*_peak_)_Test_/(*t*_peak_)_Control_ = 291 ± 46%, *n* = 6) reduced the peak amplitude by 90% (*p* = 2.1 × 10^−8^) and increased time-to-peak by 185% (*p* = 2.1 × 10^−10^); (ix) block of VGCCs (with 5 μM nicardipine) following intracellular Ca^2+^ store depletion (with 10 μM CPA), or simultaneous block of VGCCs and IP_3_Rs (with either 5 μM nicardipine / 2 μM xestospongin C or 5 μM nicardipine / 30 μM 2-APB) completely abolished [Ca^2+^]_i_ mobilization in response to CCh. Thus, significant inhibition of the CCh-induced [Ca^2+^]_i_ transient was associated with significant reduction in its rate of rise, which is indicative of the elimination of the SPCU. Altogether these results strongly suggest that following muscarinic stimulation, SPCU results from Ca^2+^ release mediated via IP_3_Rs, which are activated synergistically by (1) IP_3_ mobilized via the M_3_- G_q/11_-PLCβ pathway, and (2) Ca^2+^ entering the cell through VGCCs activated via the M_2_- G_o_- mI_cat_-membrane depolarization pathway.

### 3.4 Sub-plasmalemmal SR elements are enriched with IP_3_Rs

The above hypothesis suggests: (1) a sub-PM location of the SR elements in ileal SMCs and (2) the expression of IP_3_Rs in these SR elements.

**Fig. 8:**
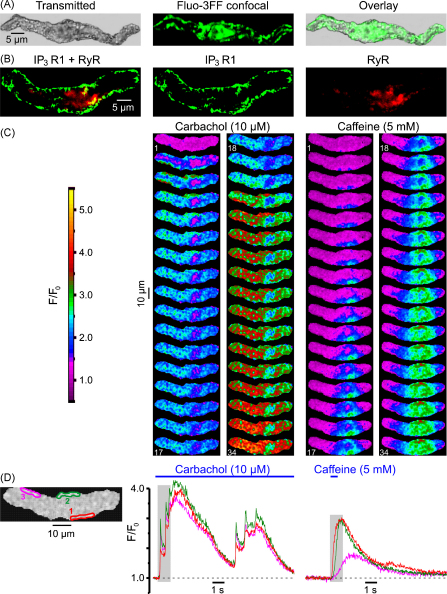
Predominant expression of IP_3_R type 1 in sub-PM SR encourages SPCU. (A) Ca^2+^ stores visualized with fluo-3FF consisted of a sub-PM SR network and some central formation. (B) Immunolocalization of IP_3_R type 1 and RyRs using a double-staining protocol (see Section 2). Confocal image of Alexa Fluor 488 fluorescence (green), showing type 1 IP_3_R distribution (middle), and confocal image of Alexa Fluor 633 fluorescence (red), showing RyR distribution (right), are overlaid (left). (C) The fluo-4-loaded SMC was stimulated with 10 μM CCh and 5 mM caffeine (with 10-min interval). Two galleries of images (acquired at 42 Hz) highlight the difference in the initial phase of the responses. (D) The temporal profiles of the fluorescence at three regions outlined in red, green and magenta (left) are shown in corresponding colour.

Intracellular Ca^2+^ stores visualized with the low-affinity (*k*_d(Ca)_ = 42 μM) Ca^2+^ indicator fluo-3FF (Fig. 8A) consisted of a sub-PM SR network and some central elements (*n* = 47). This spatial organization of intracellular Ca^2+^ stores is generally similar to that we have previously demonstrated in the rabbit portal vein myocytes using DiOC_6_ and BODIPY TR-X ryanodine [38] and SMCs of the guinea-pig mesenteric artery using brefeldin A BODIPY 558/568 (unpublished observation).

Immunodetection of IP_3_Rs (with antibody specific for type 1 IP_3_R) and RyRs (with antibody targeting type 1, type 2 and type 3 RyRs) by double labelling (*n* = 18) of the same SMC (see Section 2) revealed that type 1 IP_3_Rs are predominantly expressed in sub-PM SR elements over the entire periphery of the cell, while RyRs are absent from the ends of the SMC, but are seen in some central sub-PM and deep SR elements (Fig. 8B). Based on the results of the cell fractionation and binding studies of the longitudinal muscle layer of the guinea-pig ileum, showing that the overall stoichiometric ratio of RyRs to IP_3_Rs in SMCs from this tissue is 1:9–10, the existence of a Ca^2+^-storage compartment devoid of RyRs but equipped with IP_3_Rs has been suggested previously [39]. This is in agreement with our finding showing that only a few elements of the SR in the central region of the SMC represent a Ca^2+^ store where the type 1 IP_3_Rs and RyRs are co-expressed (yellowish spots; Fig. 8B).

Close proximity of the IP_3_R-enriched SR elements to the cell plasma membrane facilitates an abrupt IP_3_R-mediated Ca^2+^-release when [Ca^2+^]_i_ and [IP_3_]_i_ rapidly rise in the restricted microvolume between the SR and plasmalemma, as observed in the case of activation of muscarinic receptors with CCh (Fig. 8C). In contrast, when the same cell was stimulated with 5 mM caffeine, which activates RyRs to release Ca^2+^, the Ca^2+^ wave developed initially in the cell centre (where RyRs are predominant) and only then spreads to the cell periphery (Fig. 8C). The difference in the dynamics of [Ca^2+^]_i_ mobilization at three regions within the SMC following stimulation with CCh and caffeine is emphasized by the plots showing the time course of the fluo-4 fluorescence averaged within each of the three regions (Fig. 8D). In response to CCh [Ca^2+^]_i_ rapidly increased at all three regions: IP_3_R-mediated Ca^2+^ release leading to a SPCU. In contrast, in response to caffeine, RyRs released Ca^2+^ initially within the region 1, then the Ca^2+^ wave reached the region 2 and only then, with a more substantial delay, it arrived at region 3.

### 3.5 IP_3_R-mediated Ca^2+^ release is essential for force generation

**Fig. 9:**
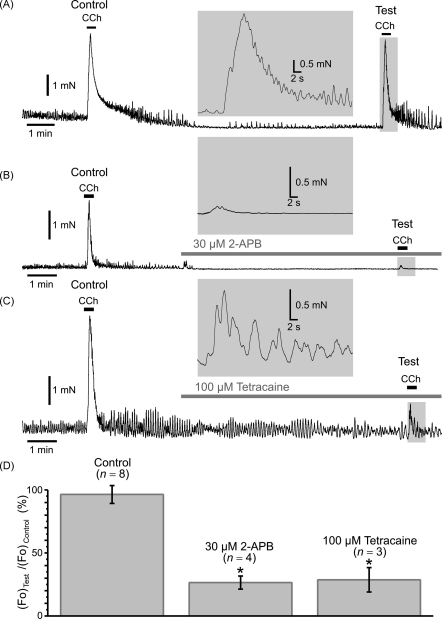
Effect of inhibition of IP_3_Rs and RyRs on isometric force of muscarinic contraction. Smooth muscle strips from longitudinal layer of the guinea-pig ileum were attached to isometric force transducer at a resting tension load of 5 mN, bathed in the PSS at 37 °C and stimulated with 2 μM CCh with a 10-min interval. The response to the second CCh application (Test) was related to the response to the first CCh application (Control). The Test response was obtained in the control (A), in 30 μM 2-APB (B) and in 100 μM tetracaine (C). Insets: the Test responses presented on an expanded time scale. A summary of the relative change in the maximal isometric force ((Fo)_Test_/(Fo)_Control_) is presented as bar diagram plot (D). The symbol (*) shows the significant difference (*p* < 0.004) between the normalized maximal isometric force in control external solution and in the presence of the drug, as indicated.

Several lines of evidence presented above strongly suggest that in SMCs freshly isolated from the guinea-pig ileum [Ca^2+^]_i_ mobilization in response to muscarinic receptor activation is initiated by a SCPU resulting from an abrupt IP_3_R-mediated Ca^2+^ release at multiple sub-PM regions where it is facilitated by Ca^2+^ entry through VGCCs. Under conditions when the SPCU is abolished (by inhibition of IP_3_Rs or VGCCs), the global [Ca^2+^]_i_ mobilization within the SMC is substantially attenuated (Fig. 7). It therefore seems likely that this IP_3_R-mediated Ca^2+^ release is a key element in the chain of events resulting in the muscarinic contractile response. To test this we examined the effect of inhibition of IP_3_Rs and RyRs on isometric force generated in response to muscarinic stimulation of a smooth muscle strip freshly dissected from the longitudinal layer of the guinea-pig ileum (Fig. 9). The strips were stimulated with 2 μM CCh, which was transiently applied to the same strip at least twice. The response to the second CCh application was referred to as the test response, while the response to the first CCh application was referred to as the control response. The test response was obtained either in the control external solution to evaluate reproducibility of the responses to CCh (Fig. 9A), or following a 7-min incubation of the strip with either 30 μM 2-APB (Fig. 9B) or 100 μM tetracaine (Fig. 9C). The maximal isometric force (Fo) detected during the test response was then normalized to that during the control response and compared in control and following incubation with the drugs (Fig. 9D). This revealed that: (i) in control external solution, the peak amplitude of the test response constituted on average 98 ± 7% (*n* = 8) of the control response; (ii) inhibition of IP_3_Rs with 30 μM 2-APB ((Fo)_Test_/(Fo)_Control_ = 26.5 ± 5%, *n* = 4) reduced the peak isometric force by 73% (*p* = 0.0006); (iii) inhibition of RyRs with 100 μM tetracaine ((Fo)_Test_/(Fo)_Control_ = 29 ± 10%, *n* = 3) reduced the peak isometric force by 70% (*p* = 0.001). This demonstrates that both IP_3_R- and RyR-mediated Ca^2+^ release are required for full scale contractile response to 2 μM CCh. However, in contrast to tetracaine, 2-APB also abolished spontaneous oscillations of isometric tension observed in control (compare Fig. 9B and C).

**Fig. 10:**
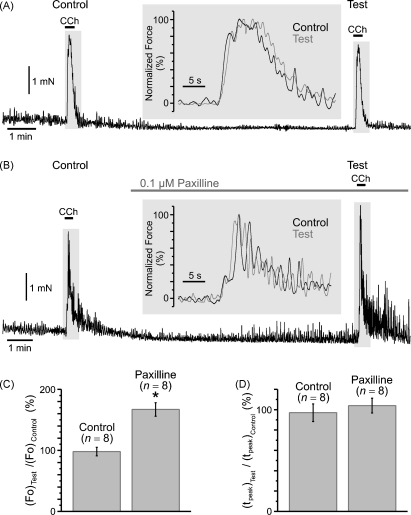
Effect of inhibition of BK channels on isometric force of muscarinic contraction. The protocol was similar to that in Fig. 9. The Test response to 2 μM CCh was obtained in control (A) and in 0.1 μM paxilline (B). Insets: the overlays of Test (black trace) and Control (grey trace) responses normalized to their maximum are presented on an expanded time scale. The bar diagram plots summarize: (C) relative change in the maximal isometric force ((Fo)_Test_/(Fo)_Control_) and (D) relative change in time-to-peak ((*t*_peak_)_Test_/(*t*_peak_)_Control_). The symbol (*) shows the significant (*p* < 0.0008) increase of the maximal cholinergic force following BK channel inhibition was not associated with any significant (*p* = 0.92) change in the kinetics of the contraction.

On the other hand, a SPCU should activate large conductance Ca^2+^-activated K^+^ channels (BK channels) in the cell membrane, which may lead to membrane hyperpolarization and, as a result, to a decrease of Ca^2+^ entry through VGCCs [11], thus slowing down [Ca^2+^]_i_ mobilization and perhaps the contractile response. This, however, was not observed. Indeed, even though the peak of the CCh-induced [Ca^2+^]_i_ transient was associated with some minor (about 8 mV) transient repolarization of the cell membrane (Fig. 1C), inhibition of IP_3_Rs with 2-APB did not accelerate the contractile response to CCh, and its kinetics remained the same in control and following 2-APB or tetracaine pre-treatment (see insets in Fig. 9A–C). Nevertheless, we tested the effect of a selective inhibitor of BK channels, paxilline [40] on CCh-induced isometric force using a similar experimental protocol (Fig. 10). Exposure of the muscle strips to 0.1 μM paxilline did not change the tonic tension, but augmented the amplitude of spontaneous oscillations of isometric tension (Fig. 10B) and increased CCh-induced isometric force ((Fo)_Test_/(Fo)_Control_ = 167 ± 11%, *n* = 8) by 70% (*p* = 0.0009; Fig. 10C). It, however, had no significant effect on the kinetics (Fig. 10D) of the muscarinic contraction: time-to-peak ((*t*_peak_)_Test_/(*t*_peak_)_Control_) in control (97 ± 9%, *n* = 8) and following paxilline treatment (104 ± 7%, *n* = 8) were not significantly different (*p* = 0.92).

## 4 Discussion

As an increase of [Ca^2+^]_i_ is a primary signal for contraction in all types of muscles, the nature of the mechanisms linking excitation to [Ca^2+^]_i_ mobilization, as well as the spatial organization and molecular composition of intracellular Ca^2+^ release units are important determinants of the contractile response. However, the mechanisms coupling excitation to [Ca^2+^]_i_ mobilization in skeletal, cardiac and smooth muscles are different. While in skeletal muscles membrane depolarization triggers Ca^2+^ release from the SR via direct interaction between the voltage sensors in the T-tubules (voltage-gated L-type Ca^2+^ channels/the dihydropyridine receptors; VGCCs) and ryanodine receptors (RyRs) expressed in the terminal sacs of the SR (reviewed in Refs. [41–43]), in ventricular cardiac muscles Ca^2+^ entry through VGCCs triggers RyR-mediated Ca^2+^ release via a Ca^2+^-induced Ca^2+^ release (CICR) mechanism (reviewed in Refs. [43–47]). In both cases the structural basis for excitation–contraction (E–C) coupling is localization of the SR RyRs at the ends of sarcomers in close juxtaposition to T-tubular VGCCs.

Cytosolic Ca^2+^ which triggers contraction of smooth muscle cells (SMCs) is mobilized either by depolarization of the cell membrane leading to Ca^2+^ entry through VGCCs (electromechanical coupling [1]), or by activation of a variety of receptors (pharmacomechanical coupling [1]) usually coupled via G_q/11_-protein to stimulation of phospholipase C (PLC), IP_3_ production and IP_3_R-mediated Ca^2+^ release, or by a combination of these mechanisms. Either of these events, which cause an initial rise in [Ca^2+^]_i_, may be further augmented by RyR-mediated Ca^2+^ release activated via CICR [5,8,16,20,34,48–52]. The relative contribution of RyRs and IP_3_Rs to intracellular [Ca^2+^]_i_ mobilization and the role of these receptors in the genesis of localized Ca^2+^-release events (sparks or puffs), propagating Ca^2+^ waves and [Ca^2+^]_i_ oscillations varies in different types of SMCs, and often depends on the strengths and mechanism of SMC stimulation. In some phasic SMCs, e.g. urinary bladder and vas deferens, Ca^2+^ entry through VGCCs is tightly coupled to RyR-mediated Ca^2+^ release. Electrical stimulations of these SMCs (under current- or voltage-clamp conditions) triggered an abrupt RyR-mediated Ca^2+^-release at multiple sub-PM regions (‘hot spots’ [5,50,53]). Using 3D-immunofluorescence, freeze fracture and thin section electron microscopy, it was demonstrated that in SMCs of urinary bladder VGCCs and RyRs are in close proximity to each other within the caveolar domains [54], thus forming a complex analogous to Ca^2+^ release units of striated muscles [55]. Destruction of caveolae in SMCs of urinary bladder with methyl-β-cyclodextrin attenuated coupling between voltage-gated Ca^2+^ entry and RyR-mediated Ca^2+^ release and reduced contractile responses elicited by electrical stimulation [52]. Nevertheless, it was demonstrated that IP_3_R-mediated Ca^2+^-release is essential for [Ca^2+^]_i_ mobilization and contraction, especially (but not exclusively) induced by stimulation of SMCs with neurotransmitters and hormones [19,20,30,33,34,56–60]. It therefore was suggested that at least in some SMC types CICR could be initiated and facilitated by IP_3_R-mediated Ca^2+^ release [16,34,48,57,58].

In this study we have demonstrated that strong muscarinic stimulation (with 10 μM CCh) of single ileal SMCs triggers an abrupt sub-PM [Ca^2+^]_i_ upstroke (SPCU) produced by IP_3_R-mediated Ca^2+^ release from sub-PM SR elements. These events were closely associated with action potentials (Fig. 1) and strongly depended on Ca^2+^ entry through VGCCs (Figs. 2A and 7), suggesting that in this SMC type E–C coupling involves an initial IP_3_R-mediated Ca^2+^ release facilitated by voltage-gated Ca^2+^ entry. A molecular basis for this is that IP_3_Rs in this cell type are expressed in much greater quantity than RyRs (an overall stoichiometric ratio of RyRs to IP_3_Rs in the guinea-pig intestinal smooth muscle was reported to be 1:9–10 [39]) and are predominantly located in sub-PM SR, while RyRs are mostly confined to the centrally located deep SR (Fig. 8B [24]), similarly to some other types of phasic SMCs [61]. Differential distribution of RyRs and IP_3_Rs explains the difference between spatio-temporal patterns of Ca^2+^ waves induced by direct stimulation of RyRs and Ca^2+^ waves triggered following IP_3_ mobilization in response to muscarinic stimulation under conditions when voltage-gated Ca^2+^ entry is either facilitated (Fig. 8C and D) or diminished (by holding the cell membrane potential at a negative level [24]).

In gastrointestinal SMCs, a mixed population of muscarinic receptors (M_2_ and M_3_) with the predominance of the M_2_ subtype (75–82%) are co-expressed (reviewed in Refs. [62–64]). There are several signal transduction mechanisms which link activation of these receptors to stimulation of IP_3_R-mediated Ca^2+^ release. The main universal Ca^2+^ signalling pathway couples activated M_3_ receptors via pertussis toxin (PTX)-insensitive G proteins (*G*_q/11_) to stimulation of phospholipase C β (PLCβ) leading to formation of IP_3_ [65,66]. In addition, activation of M_2_ receptors is coupled via PTX-sensitive G proteins (G_i_) to inhibition of adenylyl cyclase activity [63,66] leading to a decrease in cAMP level and, as a result, to suppression of the inhibitory effects of protein kinase A on the PLCβ–IP_3_–IP_3_R signalling pathway [67,68]. Finally, activation of muscarinic receptors produces excitation of gastro-intestinal (GI) smooth muscles causing depolarization of SMCs and an increase in the frequency of the action potentials via modulation of the activity of many different channel types (reviewed in Refs. [64,69,70]), of which opening of the cationic channels [35,71] synergistically regulated via M_2_ − G_αo_ [37,72,73] and/or M_2_/M_3_ − G_o_ − atypical PLC [64,74–76] and M_3_ − G_q/11_ − PLCβ − IP_3_ − Ca^2+^ [24,26,27,35–37] pathways is one of the major mechanisms of GI smooth muscle excitation. The inward Na^+^ current through muscarinic cationic channels (in many smooth muscles, including guinea-pig ileum, these channels have very low, if any, permeability to Ca^2+^ [25–27], see also Figs. 3Bc, 4d, and 6Cc) produces membrane depolarization resulting in an increased Ca^2+^ influx via VGCCs. There is growing evidence that Ca^2+^ entering SMC is “trapped” between the SR and opposed regions of the plasmalemma [59,77] resulting in an increase of the local [Ca^2+^]_i_ up to the order of 10 μM [9,77]. Using two-photon flash photolysis of “caged” Ca^2+^ it was recently demonstrated that a local increase of [Ca^2+^]_i_ may trigger IP_3_R-mediated Ca^2+^ release [51].

The SPCU triggered by stimulation of SMCs of the guinea-pig ileum with 10 μM CCh was virtually abolished by either block of VGCCs (Fig. 3A) or by depletion of intracellular Ca^2+^ stores (Fig. 3B). Thus, similarly to Ca^2+^ mobilization at sub-PM ‘hot spots’ evoked by electrical stimulation of SMCs from urinary bladder and vas deferens [5,50–52], SPCU results from the SR Ca^2+^ release induced by Ca^2+^ entry (CICR) through VGCCs. After Ca^2+^ store depletion the residual CCh-induced [Ca^2+^]_i_ transient was spatially uniform, which is characteristic of Ca^2+^ entry through VGCCs [8,50,59,77]. Indeed, subsequent inhibition of VGCCs (while keeping Ca^2+^ stores depleted) completely abolished [Ca^2+^]_i_ mobilization in response to CCh (Fig. 3B), which also demonstrates that muscarinic cationic channels in SMCs of the guinea-pig ileum are virtually impermeable to Ca^2+^ (see also [27]).

In contrast to that reported in SMCs from urinary bladder [50] (where RyRs are predominantly expressed in sub-PM SR elements [53] in close juxtaposition to plasmalemmal VGCCs within multiple caveolar domains [54]), the SPCU in SMCs of the guinea-pig ileum (where sub-PM SR elements are enriched with IP_3_Rs; Fig. 8B and [24]) was not affected by block of RyRs with either 50–100 μM tetracaine (Fig. 4) or 100 μM ryanodine (summarized in Fig. 7). Inhibition of RyRs, however, abolished the sustained phase of the CCh-induced [Ca^2+^]_i_ transient and/or [Ca^2+^]_i_ oscillations (Fig. 4), thus indicating that Ca^2+^ release via RyRs (predominantly expressed in centrally located SR in this cell type; Fig. 8B and [24]) is also involved in CCh-induced [Ca^2+^]_i_ mobilization. However, in contrast to SMCs from urinary bladder and vas deferens [5,50,53], RyR-mediated Ca^2+^ release in ileal SMCs is ‘loosely coupled’ [49,78] to Ca^2+^ entry through VGCCs. It is also evident that in ileal myocytes IP_3_Rs alone (Fig. 4b) may account for Ca^2+^ wave propagation, not unlike the situation in guinea-pig colonic myocytes [19].

In contrast with Ca^2+^ ‘hot spots’ elicited by membrane depolarization in urinary bladder SMCs, which were insensitive to inhibition of IP_3_Rs with 3 μM xestospongin C [50], CCh-induced SPCU in ileal myocytes was virtually abolished by inhibition of IP_3_Rs (Figs. 4 and 6B). It should be noted, however, that in both cell types an initial global [Ca^2+^]_i_ transient elicited by muscarinic stimulation was substantially attenuated by IP_3_R inhibition, but only in ileal SMCs this also suppressed the sustained rise of [Ca^2+^]_i_ (Fig. 6C). On the other hand, in ileal SMCs, block of VGCCs or cumulative block of VGCCs and RyRs reduced CCh-induced [Ca^2+^]_i_ transients by 90% (Fig. 7B). Altogether these observations strongly suggest that following muscarinic receptor activation: (1) SPCU results from IP_3_R-mediated Ca^2+^ release facilitated by Ca^2+^ entry through VGCCs, (2) Ca^2+^ mobilized upon the initial [Ca^2+^]_i_ transient activates RyRs to release Ca^2+^ and (3) the sustained rise of [Ca^2+^]_i_ and/or [Ca^2+^]_i_ oscillations are the result of interplay between IP_3_Rs and RyRs.

It should be noted that pharmacological agents used in this study to assess the contribution of IP_3_Rs to SPCU may affect voltage-gated Ca^2+^ entry induced by muscarinic receptor activation, namely: (1) xestospongin C was shown to inhibit barium current through VGCCs in guinea-pig ileal myocytes but had no effect on mI_cat_ [79], (2) 2-APB was reported to inhibit mI_cat_ in SMCs from murine stomach [80]. We found that 2 μM xestospongin C inhibited *I*_Ca_ by 74% (Fig. 5) and, thus, its effect on muscarinic [Ca^2+^]_i_ mobilization may partially result from inhibition of VGCCs. Nevertheless, suppression of SPCU by 30 μM 2-APB, which did not inhibit VGCCs (Fig. 5) and had no effect on CCh-induced membrane depolarization (Fig. 6B), confirms that this event results from IP_3_R-mediated Ca^2+^ release.

Under conditions when SPCU is abolished (by inhibition of IP_3_Rs or VGCCs), the global [Ca^2+^]_i_ mobilization within the SMC is substantially attenuated (Fig. 7). It therefore seems likely that SPCU is a key element in the chain of events resulting in the muscarinic contractile response. Indeed, isometric force generated by smooth muscle strips of the longitudinal layer of the guinea-pig ileum in response to 2 μM CCh was attenuated by 73% following inhibition of IP_3_Rs (Fig. 9D), similarly to that reported in the guinea-pig distal colon [30]. However, similarly to that reported in urinary bladder, where contraction evoked by electrical stimulation [50] or stimulation of muscarinic receptors [81] was shown to depend on RyR-mediated Ca^2+^ release activated by CICR, muscarinic contraction of the longitudinal layer of the guinea-pig ileum was attenuated by 70% following inhibition of RyRs (Fig. 9D). This suggests that the sustained phase of the CCh-induced [Ca^2+^]_i_ transient, which depends on RyR-mediated Ca^2+^-release (Fig. 4), is crucial for force generation and that the initial IP_3_R-mediated Ca^2+^ release serves to link Ca^2+^ entry through VGCCs to RyR-mediated Ca^2+^ release. It is noteworthy that inhibition of IP_3_Rs also abolished spontaneous oscillations of isometric tension observed in control (Fig. 9B). This indicates that IP_3_Rs are also involved in spontaneous rhythmical contractile activity of gastro-intestinal smooth muscles, which is driven by interstitial cells of Cajal (reviewed in Ref. [82]). The importance of IP_3_R type 1 for normal activity of gastro-intestinal smooth muscles was recently illustrated by the demonstration that gastric smooth muscle from mutant mice lacking the type 1 IP_3_R revealed no slow wave activity and had attenuated muscarinic excitatory responses [56].

Another important target for sub-PM Ca^2+^ mobilization in SMCs are Ca^2+^-sesitive membrane ion channels [8,9,11,33,83–85]. We have recently demonstrated that mI_cat_ in ileal SMCs is synergistically potentiated by Ca^2+^ and IP_3_ [24]. Hence, on the one hand, SPCU serves to accelerate membrane depolarization and Ca^2+^ influx through VGCCs, thus providing a positive feedback mechanism whereby Ca^2+^ entry and Ca^2+^ release promote membrane depolarization and further Ca^2+^ influx, termed Ca^2+^-induced Ca^2+^ entry (CICE) [86]. On the other hand, activation of BK channels by SPCU should limit the rate and the extent of muscarinic excitation. Nevertheless, neither inhibition of IP_3_Rs with 2-APB (which eliminates SPCU) nor direct inhibition of BK channels with 0.1 μM paxilline accelerated the contractile response to CCh (Figs. 9 and 10). Paxilline (0.1 μM), however, augmented the muscarinic contractile response by 70% (Fig. 10B and C) but had no effect on tonic tension before CCh application (Fig. 10B). A likely explanation is that Ca^2+^ entry though VGCCs facilitated by inhibition of BK channels (via the resulting greater membrane depolarization) does not alter [Ca^2+^]_i_, but instead it increases the SR Ca^2+^ load, thus causing an increase of CCh-induced isometric force. Thus, it seems likely that overall physiological effect of SPCU is to engage a rapid full-scale [Ca^2+^]_i_ mobilization, rather than to control contractile activity of intestinal smooth muscles via modulation of the BK channel activity.

In conclusion, SPCU is caused by an abrupt IP_3_R-mediated Ca^2+^ release from sub-PM SR elements facilitated by Ca^2+^ influx through VGCCs and serves to augment intracellular Ca^2+^ mobilization via CICE (acting on muscarinic cationic channels) and via CICR (acting on RyRs) mechanisms, and represents the key element in the chain of the signalling events in cholinergic contraction.
